# Xeroderma Pigmentosum: a 12-year experience in digital dermoscopy and reflectance confocal microscopy follow-up at a Cancer Center in Brazil^؆^

**DOI:** 10.1016/j.abd.2025.501182

**Published:** 2025-08-13

**Authors:** Joyce Gouvêa Freire, Tatiana Cristina Moraes Pinto Blumetti, Rafaela Brito de Paula, Juliana Casagrande Tavoloni Braga

**Affiliations:** AC Camargo Cancer Center, São Paulo, SP, Brazil

**Keywords:** Xeroderma pigmentosum, Dermoscopy, Reflectance confocal microscopy, Malignant melanoma

## Abstract

**Background:**

Xeroderma pigmentosum (XP) is an autosomal recessive genetic disorder characterized by a defect in the nucleotide excision repair (NER) pathway, responsible for repairing DNA damage induced by ultraviolet rays. The most common symptom in affected patients is an increased photosensitivity associated with early development of cutaneous and internal malignancies.

**Objective:**

To describe whether the follow-up of xeroderma pigmentosum patients using total body mapping (TBM) with digital dermoscopy (DD) and *in vivo* reflectance confocal microscopy (RCM) increases early detection of melanoma and reduces unnecessary biopsies of benign melanocytic lesions.

**Methods:**

Twelve XP patients were followed-up with TBM and DD from February 2008 until March 2020. The number of melanocytic lesions excised (NNE) was counted before and after the surveillance with TBM, DD, and RCM.

**Results:**

In the 12-year surveillance period, twelve XP patients were followed-up with TBM, DD, and RCM. The proportion of thinner and *in situ* melanomas diagnosed increased after the implementation of TBM and DD in the follow-up of this group (from 67% to 82%). The association of technologies caused a reduction in the NNE from 4.02 to 2.88 and promoted early detection of melanoma.

**Study limitations:**

Maintaining regular follow-up with some XP patients can be challenging due to comorbidities and social issues. Although XP is a rare disease, this represents an especially small number of cases.

**Conclusion:**

XP patients are generally submitted to multiple surgical excisions, with high morbidity. Based on this experience, TBM, DD and RCM have improved the early detection of melanoma and reduced the NNE with a positive impact on health and quality of life.

## Introduction

Xeroderma pigmentosum (XP) is an autosomal recessive genetic disorder characterized by a defect in the nucleotide excision repair (NER) pathway. The NER pathway is responsible for repairing DNA damage induced by ultraviolet, thus the most common symptom in affected patients is an increased photosensitivity associated with early development of cutaneous and internal malignancies.^1^

The disease is comprised of eight complementation groups (A to G) with defects in different steps of the NER pathway, and an XP variant V (XP-V), which has normal repair of photoproducts but defective DNA polymerase *n* (pol *n*).^1^ Multiple skin cancers are more common in XP-C, XP-E, and XP-V groups.^2^ The incidence of XP varies among different countries in the world: in Japan, the incidence is 1 in 20,000 to 40,000 live births, while in the United States and Europe is 1 in 250,000 to 1,000,000 live births.^3^

The clinical manifestations of XP are predominantly cutaneous and ocular, but neurologic findings are reported in a few cases.^1^ The photosensitivity is the first skin manifestation and occurs in 50% of the patients. It is characterized by exaggerated sunburn (with blisters and prolonged erythema) with minimal UV exposure.^4^ Freckling is usually seen within the first two years of age.^1^ Hyperpigmented and hypopigmented macules, telangiectasias and premature photoaging are also seen in childhood.

Early diagnosis and sun protection measures are crucial for the survival of XP patients. Individuals who are less than 20 years old have a 10,000-fold increased risk of developing non-melanoma skin cancers and a 2,000-fold increased risk of melanoma (MM) when compared to the general population. The median age of the onset of non-melanoma skin cancer is at 9 years and for the first melanoma is 22 years.^1^

It has been reported that dermoscopy significantly increases the sensitivity and specificity of the diagnosis of melanoma compared with naked-eye examination.^5^ However, in XP patients with a high number of actinic lesions (solar lentigos, lentigines and solar elastosis), the early recognition of malignancies can be challenging.^6^

With the goal to improve follow-up for patients with XP, few reports showed that digital dermoscopy could be useful. Salerni et al suggested that the use of baseline and follow-up photographs of the skin (total-body photography) combined with digital dermoscopy increases the diagnostic accuracy of malignant melanoma in high-risk patients, including those with genodermatoses like XP. In that cohort, more than 600 patients were evaluated, although only 3 were XP.^7^ In 2005, Malvehy et al. described the dermoscopy of skin lesions in two XP patients, and in 2009 Green et al. reported the follow-up of a 39 y.o. woman with XP using total-body examination and photography and, dermoscopy.^6^, ^8^

*In vivo* reflectance confocal microscopy (RCM), a noninvasive imaging technique with cellular resolution, enables the early detection of melanoma with a lower rate of excisions.^9^ It has already been proved that RCM improves the diagnostic accuracy in skin cancer detection when combined with dermoscopy, especially for difficult-to-diagnose lesions.^9^, ^10^, ^11^, ^12^ In 2019, Rocha et al first published the use of RCM on dermoscopic suspicious lesions, improving the diagnostic accuracy of malignancy in XP patients.^13^

Here the authors describe the experience in the follow-up of twelve XP patients at the Department of Cutaneous Oncology of the AC Camargo Cancer Center, São Paulo, SP, Brazil based on total-body photography associated with digital dermoscopy and the use of RCM as a complementary exam to avoid unnecessary excisions.

## Objective

To describe whether the follow-up of xeroderma pigmentosum patients using total body mapping (TBM) with digital dermoscopy (DD) and in vivo reflectance confocal microscopy (RCM) increases early detection of melanoma and reduces unnecessary biopsies of benign melanocytic lesions.

## Methods

**Figure 1:**
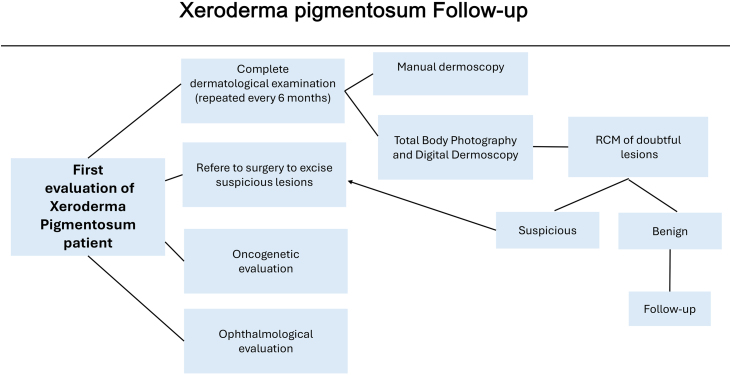
Fluxogram of the xeroderma pigmentosum patients.

Twelve XP patients were retrospectively retrieved from the Department of Cutaneous Oncology of the AC Camargo Cancer Center, São Paulo, SP, Brazil. They had been followed up with total-body photography and digital dermoscopy from February 2008 until March 2020. The study was approved by the AC Camargo Cancer Center Ethics Committee (CEP 1524/11). These patients were also referred to a multidisciplinary team of oncology surgeons, oncogenetic evaluation and ophthalmologic evaluation if necessary (Fig. 1).

The follow-up was performed every 6 months during regular dermatology clinics. The dermatological visits comprised complete dermatological examination using a manual dermatoscope (DermLite® DermLite LCC, California) as well as Total-Body photography and digital dermoscopy using Fotofinder® (Fotofinder Systems GmbH, Germany).

In a first step, previous and current total-body images were compared, and changes in color, shape, and size of pigmented lesions were evaluated, and the search for new lesions was performed. The second step consisted of the evaluation of digital dermoscopy images; the melanocytic lesions previously recorded were compared to the current images, and new observed lesions were included. Pre-existing lesions or new lesions that showed asymmetry, multiple colors and at least one of the dermoscopy suspicious features associated with MM by dermoscopic pattern analysis were included.^14^ In lesions under surveillance, increasing in size, changes in color and appearance of the following dermoscopy features were considered suspicious: peppering or multiple brown or dark globules with irregular shape or distribution, prominent pigment network, blotches, and structureless areas. The melanocytic lesions included in the digital dermoscopy records that presented atypical dermoscopic features but no changes from the previous images were entitled to follow-up. New lesions with atypical features and the ones with subtle changes were considered suspicious and referred to RCM, while melanocytic lesions with specific dermoscopic features of melanoma were excised (as described in Pattern Analysis).^14^ All the steps were performed by certified dermatologists, without the use of the Fotofinder® artificial intelligence-based system.

RCM examination was performed by an experienced dermatologist using the Vivascope 1500**®** confocal microscope (Lucid-Tech, Rochester, New York, USA). The RCM evaluation was based on features previously described for melanocytic lesions in the general population following the Brazilian Portuguese consensus terminology and translated to English for publication purposes.^9^, ^10^ The RCM confocal criteria indicative of melanoma suspicion included the presence of disarranged epidermal pattern and pagetoid cells in the epidermis, non-edged papillae, cellular atypia at the dermoepidermal junction, atypical nests, and bright nucleated cells in the upper dermis.^9^, ^10^

The number needed to excise (NNE) was calculated by dividing the total number of excised melanocytic lesions by the total number of melanomas, regardless of whether they underwent RCM evaluation or not.

## Results

During the 12years of surveillance, total-body photography and digital dermoscopy were performed with a median of 6 exams per patient (range: 2‒13). Three of the 12 patients have done only two total-body photographs and digital dermoscopies during the follow-up.

Five patients had their molecular mutation identified: three females in DNA repair gen XP-C (C.2251-1G>C), one female in XP-C (C.2251-1G>C and nonsense mutation c.1969G>T) and one male in XP-V (C.1221-1224del; T408LfsX36) (Table 1).

**Table 1:** Melanoma diagnosis and mutation profile.

Patient	Age at first DD	MM *in situ*		MM < 1 mm		MM > 1 mm		Total of melanoma	Mutation
		Before DD	After DD	Before DD	After DD	Before DD	After DD		
1	26	0	2	0	0	0	0	2	XP-C (C.2251-1G>C)
2	18	1	23	2	4	1	0	31	XP-C (C.2251-1G>C and nonsense mutation c.1969G>T
3	54	2	1	3	1	0	1	8	XP-V (C.1221-1224del; T408LfsX36
4	41	4	9	0	0	0	0	13	NT
5	13	0	4	0	1	0	0	5	XP-C (C.2251-1G>C)
6	7	0	0	0	0	0	0	0	NT
7	41	8	7	0	2	1	1	19	NT
8	8	2	3	0	0	0	1	6	NT
9	27	2	0	1	0	1	0	4	NT
10	9	0	0	0	0	0	0	0	NT
11	37	6	2	4	0	0	0	12	XP-C (C.2251-1G>C)
12	39	33	5	14	1	1	0	54	NT
**Total**		**58**	**56**	**24**	**9**	**4**	**3**	**154**	

DD, Digital Dermoscopy; MM, Melanoma; NT, Not Tested.

Among the 12 XP patients, 6 (50%) were female and 2 were from the same family (sister and brother). The mean age at the first exam with total-body photography and digital dermoscopy was 27 y.o. (range: 7‒54 y.o.). The youngest and the oldest patients who underwent digital dermoscopy were 7 and 54 y.o., respectively.

In the present data, all patients developed skin cancer before 11 y.o.; the youngest one developed a basal cell carcinoma (BCC) at 4 years of age while her first melanoma was diagnosed at 15 y.o.. The youngest patient with melanoma diagnosed during the follow-up was a 7 y.o. male.

Eleven patients had a previous history of surgical removal of squamous cell carcinoma (SCC) (n = 33) and/or basal cell carcinomas (n = 254) and eight patients of melanoma (n = 88) before the first total-body photography and digital dermoscopy. Fifty-eight (67%) of all excised malignant melanomas were *in situ,* 24 (28%) were classified as thin melanoma (Breslow ≤ 1 mm) and 4 (5%) were thick melanoma (Breslow > 1 mm). The most frequent histopathological subtype found was superficial spreading melanoma and the highest Breslow thickness was 1.55 mm. The melanomas were predominantly localized on the trunk (n = 33), followed by upper limbs (n = 24), lower limbs (n = 22) and head and neck (n = 8) (Table 1).

During the follow-up, based on total-body photography and digital dermoscopy 187 BCC, 51 SCC and 68 MM were excised. Among the excised melanomas, 56 (82%) were *in situ*, 9 (13%) with Breslow <1 mm, one with Breslow 1.8 mm, one with Breslow 8 mm (desmoplastic subtype), and the other was a primary dermal melanoma. No melanoma metastases were seen. Before the follow-up period 67% (n = 58) of all melanomas excised were *in situ* and after, the value increased to 82% (n = 56).

**Figure 2:**
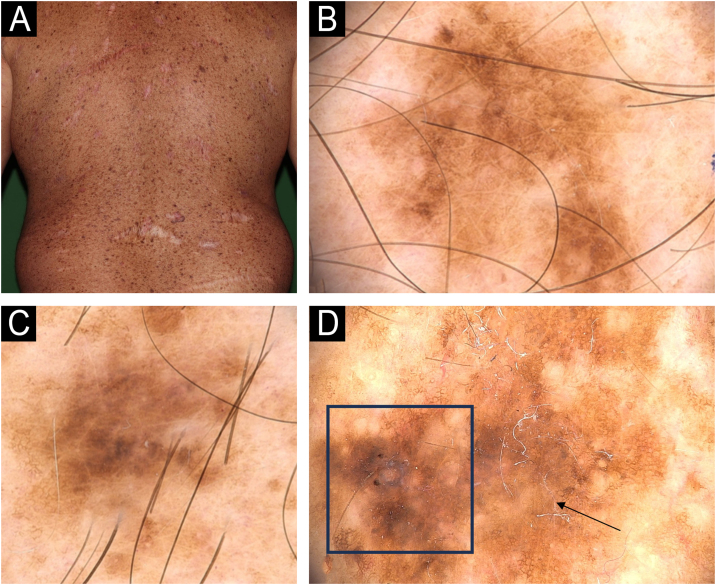
Dermoscopy patterns of benign and malignant melanocytic lesions observed in a XP patient. (A) Clinical aspect of a skin torso showing multiple freckles and pigmented lesions. (B and C) Dermoscopy of pigmented lesions with lentiginous reticular homogeneous pattern. (D) Dermoscopy of a melanoma *in situ* showing atypical, pigmented network, structureless area (arrow), hyperpigmented excentric area (blotch) with granularity (square).

**Figure 3:**
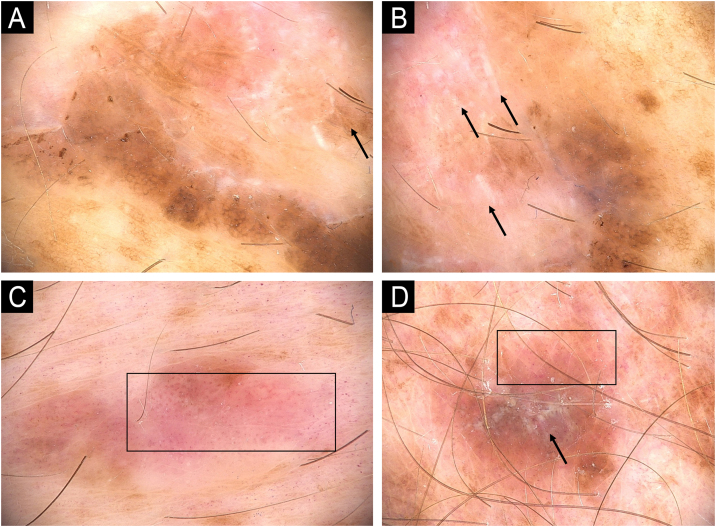
Melanoma in a XP patient. (A and B) Melanoma *in situ*: Dermoscopy showing hyperpigmented structureless areas at the periphery of the lesions and shiny-white streaks in the center (arrows). (C) Melanoma *in situ*: Hypopigmented structureless lesion showing atypical and dotted vessels in a milky red area (rectangle). (D) Superficial spreading melanoma, Breslow 0.4 mm showing shiny-white structures (arrow) and dotted vessels (rectangle).

**Figure 4:**
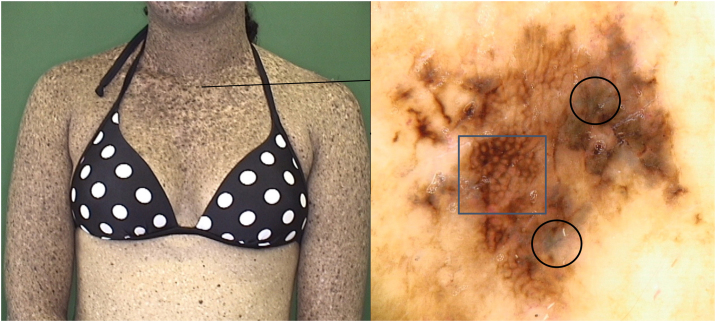
Melanoma *in situ* in a XP patient: Dermoscopy showing atypical network (square) and blue-white veil (circles).

**Figure 5:**
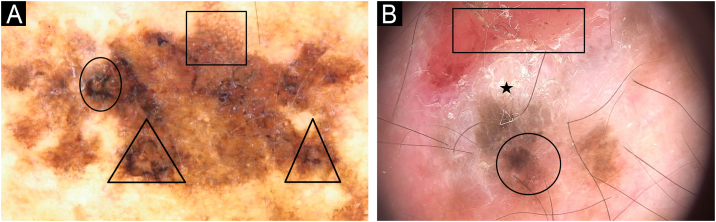
Melanoma *in situ*: (A) Dermoscopy showing atypical network (square), rhomboidal structure (circle) and angulated lines (triangle)(B) Desmoplastic melanoma: ulceration (rectangle), hyperkeratosis (star) and hyperpigmented excentric area (circle).

**Figure 6:**
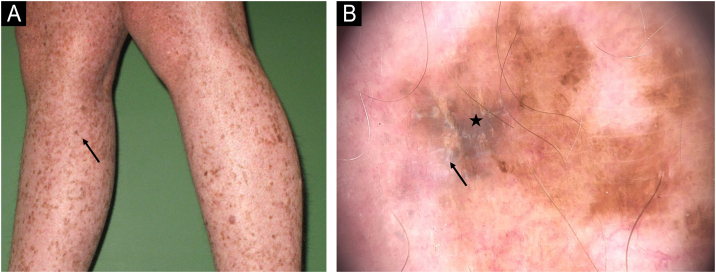
(A) Melanoma *in situ* on the right leg in a 15-year-old girl. (B) Dermoscopy showing shiny-white streaks (arrow) and blotch (star).

The dermoscopy features found on invasive melanomas were: atypical, pigmented network (90%), blotches (40%), irregular globules (30%), blue-whitish veil (30%), dotted vessels (30%), polymorphic vessels (20%), structureless areas (10%), shiny-white streaks (10 %) and milky-red areas (10%) (Table 1; Figs. 2‒6).

**Figure 7:**
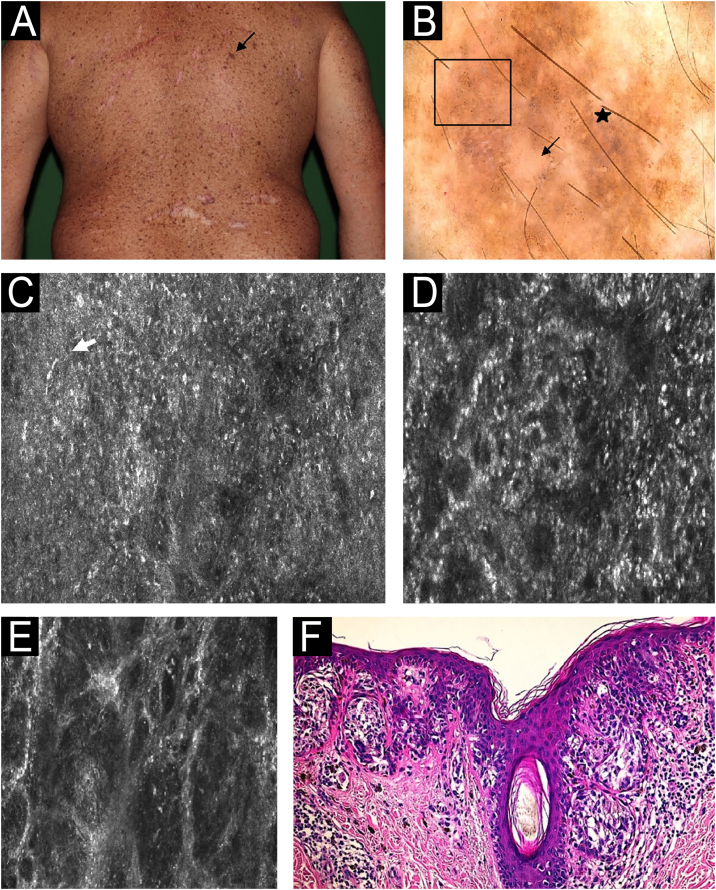
Melanoma *in situ* on the back in a 40-year-old man. (A) Clinical image: pigmented lesion on a photodamage skin (black arrow). (B) Dermoscopy: atypical dots and globules (square), peppering (star), structureless area (arrow). (C) Reflectance Confocal Microscopy (RCM); individual image (0.5 × 0.5 mm) of the spinous-granular layer. Disarranged pattern, presence of dendritic nucleated cells (white arrow). (D) RCM individual image (0.5 × 0.5 mm) of the dermoepidermal Junction (DEJ) area showing ringed pattern (center). (E) RCM at the DEJ individual image (0.5 × 0.5 mm) at the dermis showing atypical meshwork and non-edged papillae. (F) Histopathology: nested atypical melanocytes growing along dermoepidermal junction with pagetoid spread, involvement of adnexal structures and absence of dermal invasion. These microscopic features allow to confirm the diagnosis of Melanoma *in situ* (hematoxylin and eosin, 200×).

During the XP patient surveillance, lesions with small changes in dermoscopy and the new ones showing atypical patterns without melanoma features were evaluated under RCM. The features described for melanoma in the general population were applied. Disarranged pattern and the presence of dendritic nucleated cells at the spinous-granular layer, areas with non-edged papillae and dermis with atypical meshwork were the most common features in malignant lesions in the patients (Fig. 7). A total of 48 lesions were evaluated under RCM. Of these, 23 were excised, while the remaining lesions were kept under surveillance. Among the excised lesions, 16 (69%) were malignant, including 14 melanomas, 1 BCC and 1 SCC. Histological analysis of the 7 benign lesions revealed 5 cases of atypical intradermal melanocytic hyperplasia and 2 dysplastic junctional nevi.

Over the 12-year follow-up period, a total of 503 lesions were excised, 197 of which were benign. Among these benign lesions, 128 were melanocytic, while 69 were nonmelanocytic, including seborrheic keratosis and actinic keratosis. In the current study, the NNE found was 2,88. However, when the patients were evaluated using RCM, a significant improvement was observed, reducing the NNE to 1.64.

## Discussion

Xeroderma pigmentosum is a rare genetic inherited disorder caused by a defect in UV-induced DNA repair (nucleotide excision repair or translesion synthesis).^15^ Mutations in eight different genes have already been identified in XP patients, from which seven (XPA-XPG) are associated with a defect in nucleotide excision repair and one (XP-V) which codes a defective DNA polimerase *n*.^16^, ^17^ Therefore, photo-protection is the only imperative measure to avoid the development of sunburn and skin cancers.

XP-C, XP-E and XP-V groups are characterized by normal sunburn reactions. In XP-C patients, the first symptoms are photo-distributed lentigines around the age of 2 y.o. Due to the absence of exacerbated sunburns, they generally have a later age diagnosis and thus accumulate more photo-damage, leading to an earlier age of first skin cancer.^18^, ^25^ Among the four XP-C patients in the present study, the earliest skin cancer diagnosed was at the age of 4 y.o.

XP-V patients tend to be diagnosed much later, with patients living two decades or more without symptoms. Consequently, they accumulate more UVR-induced mutations and can develop hundreds of skin tumors later in life.^2^ The XP-V patient in the data developed his first carcinoma and melanoma at the ages of 24 y.o. and 43 y.o, respectively. This is the oldest patient, aged 60 and who has already had removed five melanomas and ninety-three carcinomas. This data is similar to the literature about XP-V, in which symptoms occur later, and the patients have a longer life expectancy than the other groups.^19^

The skin of these patients was characterized by the presence of a great number of solar lentigines and poikiloderma on sun-exposed areas. This phenotype adds a challenge to the early diagnosis of skin cancers, even for an experienced dermatologist, either because of the presence of an extensive number of lesions with delicate pigment network from lentigines as well as the telangiectasia due to poikiloderma that can mimic the arborizing vessels described in BCC.^6^

Regarding melanocytic lesions, the majority of patients presented lentiginous lesions and the absence of common acquired (junctional, compound, dermal) and congenital nevi. These findings were similar to the study by Malvehy et al., which described phenotypic aspects from two siblings with XP.^6^ Massaki et al. compared melanocytic lesions from XP and non-XP patients and showed histological differences: XP nevi are characterized by a lentiginous proliferation of single unit or small nests of melanocytes along the dermal-epidermal junction and often show attenuation of rete ridges, in contrast to the junctional nevi seen in general population which show elongated rete ridges with small nests of melanocytes along the tips and sides of the rete ridges.^18^ This explains the dermoscopy pattern that the authors found in the follow-up during these years.

The most common dermoscopy feature observed in invasive melanomas was an atypical pigmented network, which was seen in 90% of cases. The blue-white veil was seen in 30% of cases, and shiny-white lines, described as associated with invasive melanoma by Lallas et al. and Silva et al. were observed in 10% of invasive melanomas.^26^, ^27^ More recently, Polesie et al. observed that shiny-white lines and atypical blue-white structures were both associated with a Breslow thickness >1 mm.^28^ In the present findings, unlike what is reported in the literature, shiny-white lines were frequently observed in melanoma *in situ*, while blue-white structures were only associated with Breslow greater than 1 mm.

Total-body photography and digital dermoscopy were shown to improve the surveillance of high-risk groups by providing the benefit of not overlooking featureless melanoma and reducing the unnecessary excision of benign lesions.^5^ In addition, in XP patients, sun-damaged skin and high density of light to dark brown pigmented lesions, dermoscopy allows for the discrimination between the delicate pigment network of the multiple actinic lentigo from the true pigment network characteristic of melanocytic lesions. The digital follow-up in 3-, 6- or 12-months intervals that had already been proved to be effective for other high-risk groups, and in our opinion, is also effective in XP patients. Previous descriptions regarding the follow-up of XP patients using digital dermoscopy have already been published, although all of them have involved a small number of studied patients.^6^, ^7^, ^8^, ^13^, ^22^

In an attempt to improve the diagnostic accuracy in skin cancer detection, even for difficult-to-diagnose lesions and to avoid unnecessary excisions, RCM was performed in association with digital dermoscopy. During the XP patient surveillance, lesions with small changes in dermoscopy and the new ones showing atypical patterns without melanoma features were evaluated under RCM. The features described for melanoma in the general population were applied.^9^, ^10^, ^29^

The present study showed an improvement in diagnosing melanoma at earlier stages after the implementation of total body mapping, digital dermoscopy, and reflectance confocal microscopy in the XP patients (from 67% to 82% of melanomas *in situ*). The association of this cellular resolution technology as a complementary exam in doubtful lesions allowed for earlier melanoma diagnosis and avoided unnecessary excisions. Recently, Rocha et al showed 85.7% sensitivity and 46.8% specificity for melanoma diagnosis in XP patients using RCM.^12^ This result validates the need for close and specialized follow-up of these high-risk patients by a specialized center with the aim of reducing the risk of advanced disease and mortality.

The NNE is an effective method to measure the accuracy in melanoma detection and has decreased significantly over time in specialized clinics dedicated to skin cancer because of the routine use of diagnostic techniques such as digital dermoscopy and reflectance confocal microscopy.^20^, ^21^ Argenziano et al showed in a 10-year multicenter survey an improvement from 12.8 to 6.8 in the NNE with the use of digital dermoscopy in the evaluation of melanocytic lesions.^19^ More recently, RCM has demonstrated to be able to reduce the number of excised lesions, reaching numbers close to 3‒6.^23^, ^24^ Despite the challenges of the follow-up in XP patients, this study found an NNE of 2.88, similar to the 2.87 previously published by Alarcon et al. using RCM and dermoscopy in general and XP patients.^12^ Prior to the adoption of digital dermoscopy and RCM, the NNE was 4.02, indicating a 28% improvement.

XP patients are generally submitted to multiple surgical excisions from an early age, with high morbidity. The combined use of total-body photography, digital dermoscopy and RCM has great potential to promote the early diagnosis of cutaneous malignancies and avoid unnecessary excisions in an attempt to enhance the prognosis and quality of life of XP patients.

## Financial support

None declared.

## Authors' contributions

Joyce Gouvêa Freire: Statistical analysis; approval of the final version of the manuscript; conception and planning of the study; drafting and editing of the manuscript; collection, analysis, and interpretation of data; critical review of the literature; critical review of the manuscript.

Tatiana Cristina Moraes Pinto Blumetti: Approval of the final version of the manuscript; conception and planning of the study; drafting and editing of the manuscript; effective participation in research orientation; critical review of the literature; critical review of the manuscript.

Rafaela Brito de Paula: Drafting and editing of the manuscript.

Juliana Casagrande Tavoloni Braga: Approval of the final version of the manuscript; conception and planning of the study; drafting and editing of the manuscript; effective participation in research orientation; critical review of the literature; critical review of the manuscript.

## Data Availability

The entire dataset supporting the results of this study was published in this article.

## Scientific Editor-in-Chief

Sílvio Alencar Marques.

## Conflicts of interest

None declared.
